# β-TrCP is dispensable for Vpu's ability to overcome the CD317/Tetherin-imposed restriction to HIV-1 release

**DOI:** 10.1186/1742-4690-8-9

**Published:** 2011-02-10

**Authors:** Hanna-Mari Tervo, Stefanie Homann, Ina Ambiel, Joëlle V Fritz, Oliver T Fackler, Oliver T Keppler

**Affiliations:** 1 Department of Infectious Diseases, Virology, University of Heidelberg, Heidelberg, Germany; 2 Department of Medicine, University of California San Diego, La Jolla, California, USA

## Abstract

**Background:**

The cellular transmembrane protein CD317/BST-2/HM1.24/Tetherin restricts HIV-1 infection by physically tethering mature virions to the surface of infected cells. HIV-1 counteracts this restriction by expressing the accessory protein Vpu, yet the mechanism of this antagonism is incompletely understood. β-TrCP is the substrate recognition domain of an E3 ubiquitin ligase complex that interacts with the di-serine motif S52/S56 in the cytoplasmic tail of Vpu to target the CD4 receptor for proteasomal degradation. Recently, it has been suggested that β-TrCP is also critically involved in Vpu's ability to overcome the CD317-mediated virion release block.

**Results:**

To test this model, we analyzed the consequences of several experimental strategies to interfere with the Vpu-β-TrCP protein-protein interaction. Under these conditions, we studied effects of Vpu on expression and localization of CD317 and CD4, as well as on its ability to promote HIV-1 release. Our results demonstrate a strict requirement for Vpu's di-serine motif for degradation of CD4 and also CD317, reduction of cell surface exposure of CD317, and HIV-1 release enhancement. We further show a critical role of β-TrCP2, but not of the structurally related β-TrCP1 isoform, for Vpu-mediated degradation of both receptors. Most importantly, Vpu remained active in downregulating CD317 from the cell surface and in overcoming the HIV-1 release restriction in β-TrCP-depleted cells.

**Conclusions:**

These results demonstrate that β-TrCP is not strictly required for Vpu's ability to counteract the CD317-imposed virion release block and support the relevance of cell surface down-modulation of the restriction factor as a central mechanism of Vpu antagonism. Moreover, we propose the existence of a critical, yet to be identified cellular factor that interacts with Vpu via its di-serine motif to alter the trafficking of the restriction factor.

## Background

HIV-1 infection and replication occur in a complex environment. The host cell deploys restriction factors to stop the spread of the virus, and the virus uses its own countermeasures to promote infection. CD317 (BST-2/HM1.24/Tetherin) is a recently discovered restriction factor that can limit virus replication. It blocks the release of a diverse spectrum of enveloped viruses, including primate lentiviruses, simple retroviruses, filoviruses, arenaviruses and rhabdoviruses [1-8]. CD317 causes mature virus particles to be retained at the cell surface (also referred to as "virion tethering") [2,9]. CD317 dimers apparently connect the virion and plasma membrane without the physical involvement of other host cell factors [10].

To overcome the restriction by CD317, HIV-1 expresses the Vpu protein, which in *cis *or *trans *rescues particle release [2,9]. However, the mechanisms and cellular pathways underlying Vpu's antagonistic activity have not been fully elucidated. Vpu antagonism might involve direct binding to the restriction factor, targeting CD317 for degradation, selective downregulation of surface-exposed CD317, its exclusion from virion incorporation or altered subcellular trafficking of the restriction factor [10-18].

Vpu also mediates degradation of the CD4 receptor and inhibits activation of the transcription factor NF-κB [19,20]. Via the substrate recognition unit beta-transducin repeat-containing protein (β-TrCP), Vpu recruits the multi-subunit SCF (Skp1/Cullin/F-box protein)-E3 ubiquitin ligase complex to the endoplasmic reticulum and bridges it to the targeted host cell protein [21]. The Vpu-β-TrCP interaction requires a canonical di-serine DS_52_GxxS_56 _motif in the cytoplasmic tail of Vpu. Of note, human cells encode two structurally related β-TrCP isoforms. β-TrCP1 and β-TrCP2 function redundantly to regulate IκBα and β-catenin homeostasis [22,23] and possibly also Vpu-mediated degradation and surface-downregulation of CD4 [24].

Based on these known interactions, several laboratories have searched for a role for β-TrCP in the interaction of Vpu and CD317. However, a number of critical issues are unresolved. First, the functional importance of the Vpu di-serine motif is controversial. Mutant analyses were inconclusive: some reported a complete loss of Vpu antagonism [12,14,25], and others observed substantial virion release rescue [15,16,26,27]. Second, although β-TrCP has been implicated in Vpu's capacity to reverse the release block, the proposed mechanisms differ considerably. Vpu might co-opt the β-TrCP/SCF-E3 ubiquitin ligase complex to induce endo-lysosomal trafficking and non-proteasomal degradation to remove CD317 from the cell surface [15,16], the presumed site of its activity as a virion-tethering factor. Alternatively, Vpu might orchestrate a β-TrCP2-dependent proteasomal degradation of CD317 [14]. Finally, β-TrCP2 might be critical for both the Vpu-mediated surface downregulation and degradation of CD317, yet only in part responsible for enhancing virion release [27]. Moreover, in a recent study, we showed that the abilities of Vpu to degrade CD317 and to enhance virion release can be genetically uncoupled [17], questioning the importance of β-TrCP-dependent degradation for Vpu antagonism.

These controversies prompted us to reassess the role of the Vpu-β-TrCP interaction for HIV-1. We used a set of Vpu serine mutants, an E3 ubiquitin ligase-binding-deficient β-TrCP deletion mutant that functions as a di-serine motif-specific Vpu inhibitor, and small interfering RNA (siRNA)-depletion of individual β-TrCP isoforms to dissect the functional importance of this protein-protein interaction. We investigated the consequences of these specific manipulations, in parallel, on the Vpu-mediated rescue of virus release and on surface and intracellular levels of CD4 and CD317.

## Results

### Vpu requires its di-serine motif to deplete CD4 and CD317 and promote virus release

**Figure 1:**
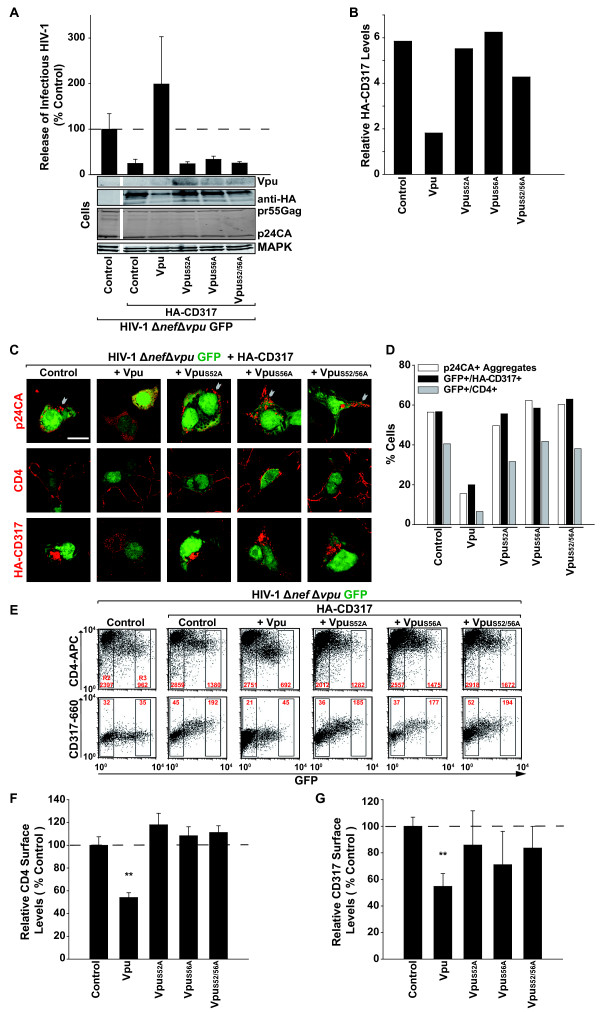
**Mutation of specific serine residues in the Vpu cytoplasmic tail cripples its ability to deplete CD4 and CD317 and to promote HIV-1 release**. 293TCD4 cells were transfected to express HIV-1Δ*nef *Δ*vpu *GFP and HA-CD317 together with either Vpu wt ("Vpu") or Vpu mutants, in which S52 or/and S56 were replaced by alanine. (A) Two days post-transfection, the yield of infectious HIV-1 in the supernatant and cell-associated levels of Vpu, HA-CD317, p24CA, and MAPK were analyzed. Western blots shown represent samples run on the identical gel with gaps indicating areas where non-informative lanes were omitted. The HIV-1 yields are plotted relative to the condition in the absence of HA-CD317 (Control), which was set to 100%. Shown are arithmetic means + SD (n = 6) from one of four similar experiments. (B) Cell-associated CD317 levels relative to MAPK are given in arbitrary units after quantification of the western blots shown in A. (C) Cells from (A) were processed for microscopic analysis to visualize p24CA^+ ^aggregates (red, upper row), CD4 expression (red, middle row) or HA-CD317 expression (red, bottom row) in transfected, HIV-expressing (GFP-positive) cells. Arrow heads indicate plasma membrane or intracellular p24CA^+ ^aggregates. Scale bar: 10 μm. Shown are representative images of four independent experiments. (D) Quantification of the frequency of cells that displayed p24CA^+ ^aggregates (white bars), co-expressed provirus-encoded GFP (+indicated Vpus) and HA-CD317 (black bars) or co-expressed GFP (+indicated Vpus) and CD4 (grey bars) in cells from the experiment shown in (C). (E-G) In cells from (A) surface levels of stably expressed CD4 and transiently expressed CD317 were monitored by flow cytometry as a function of the provirally expressed GFP and for the indicated co-expressed Vpu proteins. Panel E shows representative FACS dot plots. For quantification, the mean fluorescence intensity (MFI) for surface-exposed CD4 and CD317 was determined on highly GFP-positive cells in the R3 gate relative to the MFI of GFP-negative cells in the R2 gate (see panel (E) for gating and MFI values). MFI values obtained for control cells, not expressing Vpu, were set to 100% (panels F, G). Shown are arithmetic means ± SEM from 3 to 4 independent experiments. Student's *t*-test: **p < 0.01.

As a first experimental approach, we constructed individual and double alanine substitutions of serine residues 52 and 56 in the cytoplasmic tail of Vpu to study the role of this motif for Vpu activities. To assess multiple Vpu functions in one experimental set-up, 293T cells that constitutively express CD4 (293TCD4 cells) were co-transfected with HIV-1Δ*vpu*Δ*nef *GFP proviral DNA (an infectious provirus that carries an IRES-driven *gfp *cassette), an expression construct for CD317 carrying an N-terminal HA-tag (pHA-CD317), and expression plasmids encoding Vpu wild-type (wt) or Vpu mutants. *nef*-defective proviruses were used to allow parallel quantification of the well-established effects of Vpu on CD4 surface exposure in the absence of an additional Nef-mediated internalization of the receptor. Two days later, supernatants were analyzed for the release of infectious HIV-1 in a luminometric infectivity assay on TZM-bl reporter cells (Figure 1A), and cells were processed for Western blotting (Figure 1B), confocal microscopy (Figure 1C,D) and flow cytometry (Figure 1E-G).

Expectedly, when HA-CD317 was present during virus production, significantly less Vpu-defective HIV-1 was released (Figure 1A). Imaging by confocal microcopy showed large p24CA-positive aggregates in up to 60% of these producer cells (Figure 1C (upper row, control); quantification in Figure 1D, open bar). These aggregates most likely correspond to accumulations of tethered virions present at the cell surface or intracellularly, following their internalization [2,12]. *Trans*-expression of Vpu wt, although only barely detectable by Western blotting, readily overcame the release restriction for HIV-1Δ*vpu*Δ*nef *GFP (Figure 1A), and this effect on particle release was paralleled by a marked reduction in the fraction of cells that displayed Gag aggregates (Figure 1C (upper row image, +Vpu); Figure 1D, open bar). Furthermore and consistent with recent reports [12,13], Vpu wt expression led to a significant reduction of cell-associated levels of HA-CD317 as assessed by Western blotting (Figure 1A; quantification in Figure 1B). Similar results were obtained when scoring for the frequency of cells that displayed reduced CD317 expression levels by confocal microscopy (Figure 1C (lower row image, +Vpu), quantification in Figure 1D, filled bar), an approach that, due to its inherently lower sensitivity, frequently rendered CD317 undetecable in Vpu-expressing cells. In contrast to the wt protein, *trans*-expression of the Vpu_S52A_, Vpu_S56A_, and Vpu_S52/S56A _mutants failed to augment release of Vpu-defective HIV-1 (Figure 1A). For the di-serine mutant of Vpu, this inability to support release was confirmed in the context of a recombinant full-length HIV-1 provirus in TZM-bl cells expressing endogenous CD317 (see Additional File 1; Figure S1). In contrast to a recent report [28], no major effects of HA-CD317, Vpu, or its serine mutants, were observed for the expression levels of HIV-1 Gag or its maturation in virus-producing cells (Figure 1A, see also Additional File 2; Figure S2). Also, all three Vpu mutants did not significantly impair HA-CD317 expression (Figure 1A (Western blot); quantification in Figure 1B; Figure 1C (lower row images, + Vpu mutants), quantification in Figure 1D, filled bars), even though they displayed slightly increased steady-state levels relative to Vpu wt due to their higher stability [29]. Parallel analysis for expression of CD4 revealed the expected [19] and marked reduction of the virus binding receptor in the presence of Vpu wt, but not of its serine mutants (Figure 1C (middle row images), quantification in Figure 1D, grey bars).

Next, we assessed surface levels of CD4 and CD317 on 293TCD4 cells, which had been transiently co-transfected with HIV-1Δ*vpu*Δ*nef *GFP, pHA-CD317 and expression plasmids for the indicated Vpu proteins (Figure 1E-G). In this set-up, the provirus-driven GFP expression served as a marker for the transfection level of individual cells. The surface exposure of the stably expressed CD4 was reduced by approximately 45% on cells expressing high levels of Vpu wt (Figure 1E, upper row, third FACS plot from the left; quantification in Figure 1F), as assessed by flow cytometry. The dependence of this effect on the integrity of the di-serine motif was demonstrated by the *trans*-expression of the three Vpu mutants, none of which demonstrated downregulation of CD4 (Figure 1F).

In control 293TCD4 cells transiently transfected with pHA-CD317, an increase of surface levels of the restriction factor relative to the transfection level of these cells was noted (Figure 1E, lower row, second FACS plot from the left). Similar to what was observed for CD4 on the same cells, co-expression of Vpu wt prevented surface exposure of CD317 in a di-serine motif-dependent fashion (Figure 1E, lower row; quantification in Figure 1G). To rule out that co-expression of two cellular Vpu targets, CD4 and CD317, on the same cell affected HIV particle release or effects of Vpu on CD317 expression, we repeated the assay in parental 293T cells in the absence of CD4 and obtained comparable results (data not shown). Taken together, the Vpu di-serine motif is essential for the ability of the accessory protein to enhance HIV-1 release, to reduce surface levels of CD4 and CD317, and to trigger the depletion of both of these receptors in infected cells.

### A di-serine motif-specific Vpu inhibitor impedes Vpu's ability to downregulate cell surface CD4 and CD317 and to promote virus release, but not to prevent depletion of CD317

**Figure 2:**
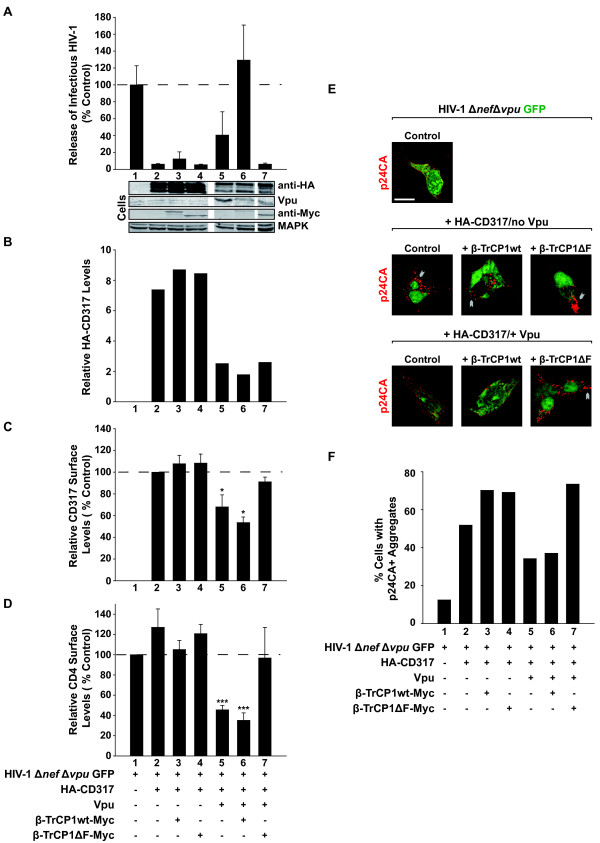
**Expression of an F-box-deficient β-TrCP impairs Vpu antagonism and surface downregulation of CD4 and CD317, but not depletion of CD317**. 293TCD4 cells were transfected to express HIV-1Δ*nef *Δ*vpu *GFP, HA-CD317, and Vpu together with either Myc-tagged β-TrCP1 wt or β-TrCP1ΔF. Two days post-transfection, supernatants and cells were analyzed as described in the legend to Figure 1. (A) The yield of infectious HIV-1 and (B) cell-associated levels of HA-CD317, Vpu, β-TrCP-Myc, and MAPK were determined. Western blots shown represent samples run on the identical gel with gaps indicating areas where non-informative lanes were omitted. (C, D) Relative surface levels of CD317 and CD4 on HIV-1-expressing (GFP-positive) cells. Shown are arithmetic means ± SEM of 3 to 6 independent experiments. Student's *t*-test: * p < 0.05, *** p < 0.001. (E, F) Cells were processed for microscopic analysis to visualize and quantify the frequency of cells with p24CA^+ ^aggregates in relation to co-expressed β-TrCP1 wt or β-TrCP1ΔF. Values are from one experiment and representative for two analyses. Arrow heads indicate plasma membrane and intracellular p24CA^+ ^aggregates. Scale bar: 10 μm.

To further probe the importance of the di-serine motif for Vpu functions, we used an F-box deletion mutant of β-TrCP1. β-TrCP1ΔF fails to connect Vpu-substrate complexes to proteasomal degradation since the F-box domain is required for β-TrCP to interact with the Skp1 adaptor of the SCF-E3 ligase complex [21,30-32]. On one hand, β-TrCP1ΔF competes with β-TrCP wt for physiological interaction partners and may consequently exert dominant-negative activities. In the context of HIV-1 infection, on the other hand, this β-TrCP1ΔF fragment functions as a motif-specific Vpu inhibitor since it efficiently binds to Vpu through the phosphorylated di-serine motif [14], blocking access of β-TrCP, but also of other putative interactors to this motif. In 293TCD4 cells transiently expressing HA-CD317, concomitant expression of β-TrCP1ΔF, but not of β-TrCP1 wt, completely abolished the Vpu-mediated rescue of virion release (Figure 2A, compare lanes 6 and 7) and restored the appearance of large p24CA-positive aggregates (Figure 2E (lower row images); quantification in Figure 2F, compare histogram bars 6 and 7). Expression of β-TrCP1ΔF also blocked Vpu's ability to downregulate CD4 from the cell surface (Figure 2D) and to deplete cell-associated CD4 levels (data not shown), as reported [21]. Importantly, co-expression of F-box-deficient β-TrCP1 with Vpu restored CD317 surface exposure to control levels observed in the absence of Vpu (Figure 2C, compare histogram bars 6 and 7), but did not prevent the Vpu-mediated depletion of intracellular pools of the restriction factor (Figure 2A, B, compare lanes 6 and 7).

**Figure 3:**
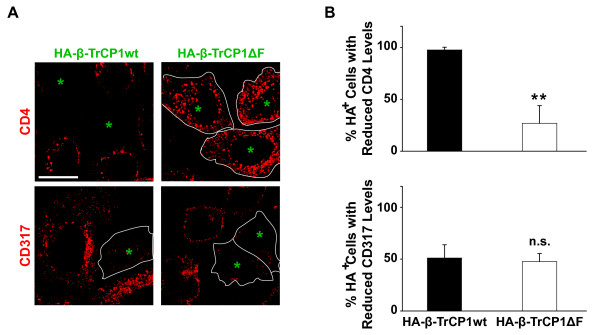
**Overexpression of an F-box-deficient β-TrCP abolishes Vpu-mediated depletion of CD4, but not of endogenous CD317**. HeLaP4 cells were transfected with expression constructs for Vpu and either HA-tagged β-TrCP1 wt or β-TrCP1ΔF. Two days later, cells were processed for microscopic analysis to (A) visualize and (B) quantify the expression of either CD4 or endogenous CD317 (both in red) in relation to HA-β-TrCP1 wt or the HA-β-TrCP1ΔF mutant (cells co-expressing HA-tagged β-TrCPs and Vpu are labelled by a green asterisk; this fluorescent channel is not depicted). The quantification in (B) depicts the percentage of cells that displayed markedly reduced levels of CD4 (upper panel) or CD317 (lower panel) in cells co-expressing Vpu together with either HA-tagged HA-β-TrCP1 wt or HA-β-TrCP1ΔF relative to untransfected neighbouring cells. The arithmetic means ± SEM of three independent experiments are shown. Scale bar: 10 μm. Student's *t*-test: ** p = 0.0021, n.s. = not significant.

Next, we sought to determine if the discordant influence of the β-TrCP1ΔF mutant on intracellular levels of CD4 and CD317 could be recapitulated in cells expressing endogenous CD317. As expected, HeLaP4 cells, expressing Vpu together with HA-β-TrCP1wt, showed a depletion of both CD4 and CD317 as assessed by confocal microscopy at the single-cell level (Figure 3A,B, Vpu and HA-β-TrCP1 wt-co-expressing cells indicated by the green asterisk). Importantly, co-expression of β-TrCP1ΔF completely abrogated Vpu's ability to deplete cell-associated levels of CD4 (Figure 3A, B, upper panels), but did not interfere with its ability to reduce levels of endogenous CD317 (Figure 3A, B, lower panels). In summary, mirroring results obtained for the Vpu_S52/S56A _mutant, expression of β-TrCP1ΔF crippled both the capacity of Vpu to promote HIV-1 release and to downregulate cell surface-exposure of CD4 and CD317. As a notable discrepancy with the di-serine mutant of Vpu, expression of β-TrCP1ΔF did not prevent depletion of intracellular pools of CD317 by Vpu, indicating that this β-TrCP1-based di-serine motif-binding fragment may induce rerouting of CD317-Vpu complexes to an alternative degradation pathway (see summary in Table 1 and Discussion).

**Table 1:** Effect of the Vpu_S52/56__A _mutant, β-TrCP1ΔF expression, or β-TrCP depletion by RNA interference on CD4 and CD317 surface levels, CD317 degradation and HIV-1 release enhancement by Vpu

	CD4	CD317		HIV-1 Release
	Downregulation^a^	Downregulation^a^	Degradation^b^	Enhancement^c^
Vpu	+	+	+	+
Vpu_S52/56A_	-	-	-	-
Vpu + β-TrCP1 wt	**+**	**+**	**+**	**+**
Vpu + β-TrCP1ΔFbox	-	-	-	-
Vpu + β-TrCP1 KD^d^	**+**	**+**	**+**	**+**
Vpu + β-TrCP2 KD^d^	-	**+**	-	**+**
Vpu + β-TrCP1/2 KD^d^	-	**+**	n.a.	**+**

^a^Vpu-mediated downregulation of CD4 and CD317 from the surface of HIV-1-infected or transfected cells was quantified by flow cytometry (Figures. 1E-G; 2C, D; 4C; 8D)

^b^Vpu-mediated depletion of CD317 in HIV-1-infected or transfected cells was analyzed by quantitative immunoblotting (Figures. 1B; 2B) and/or immunofluorescence microscopy (Figures. 1C, D; 2E-F; 3A, B; 5B, C; 8B, C)

^c^The ability of Vpu to overcome the CD317-mediated HIV-1 release restriction was analyzed by quantification of infectious virus in culture supernatants (Figures. 1A; 2A; 3A, 5A; 8A)

^d^KD: siRNA-mediated knockdown (verified by RT-PCR)

n.a.: not analyzed

### siRNA-mediated depletion of endogenous β-TrCP2, but not β-TrCP1, abrogates Vpu's ability to downregulate CD4 from the cell surface

To further characterize the requirement for endogenous β-TrCP in Vpu function, we performed RNA interference studies using siRNAs which specifically target either *β-TrCP1 *or *β-TrCP2 *mRNA. Because antibodies for reliable detection of these β-TrCP isoforms are lacking, we first validated effectiveness and specificity of these siRNAs using co-expressed Myc- or Flag-tagged β-TrCP1 or β-TrCP2 constructs (data not shown). In addition, we established a real-time PCR-based quantification of the respective endogenous *β-TrCP *mRNAs. This assay allowed us to confirm the isoform-specific interference by the siRNAs and to evaluate knockdown efficiencies in subsequent functional studies (see below).

**Figure 4:**
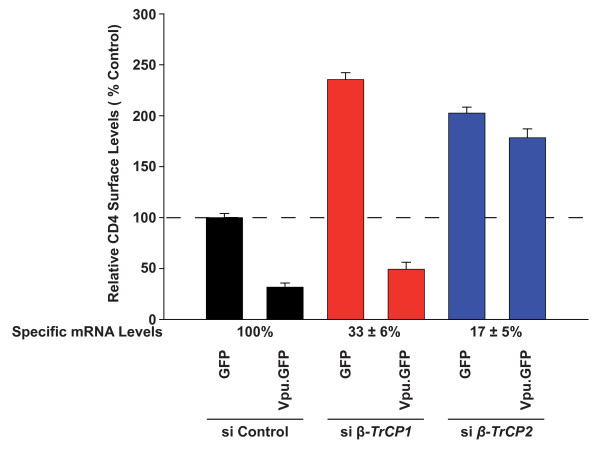
**siRNA-knockdown of β-TrCP2 abrogates the Vpu-mediated loss of CD4 from the cell surface**. 293TCD4 cells were transfected twice with the siRNAs targeting the indicated *β-TrCP *mRNAs or with a control siRNA and during the second transfection, plasmids encoding either a Vpu.GFP fusion protein or GFP alone were added. Two days post-transfection, cell-surface levels of CD4 were quantified in relation to GFP expression by flow cytometry, as reported for Figure 1E-G. *β-TrCP *mRNA levels were quantified by real-time PCR at the end of the experiment. *β-TrCP *mRNA values for control siRNA-transfected cells were set to 100%, and relative remaining levels of the specific mRNAs in knockdown cells were calculated by the 2^-ΔΔCt ^method. Shown are the arithmetic means ± SD of triplicates. Three independent experiments were performed.

In a first functional analysis, we tested the effects of siRNA-mediated depletion of endogenous β-TrCP on the ability of Vpu to downregulate surface-exposed CD4. 293TCD4 cells were transfected twice with siRNAs targeting selectively either *β-TrCP1 *or *β-TrCP2 *or with a control siRNA. During the second transfection expression constructs for Vpu.GFP or GFP were added. Of note, silencing of both *β-TrCP1 *and *β-TrCP2 *elevated steady-state levels of surface-exposed CD4 in GFP-expressing control cells by 2.4- and 2.0-fold, respectively, indicating a role for both β-TrCP isoforms in the physiological homeostasis of CD4, at least in 293TCD4 cells (Figure 4). Importantly, β-TrCP2 depletion (83% mRNA reduction) completely abrogated Vpu's ability to downmodulate CD4, while β-TrCP1-depleted cells (67% mRNA reduction) still displayed an efficient Vpu-mediated loss of CD4 surface levels (Figure 4). This finding is in contrast to a report that suggested the requirement of both β-TrCP isoforms for Vpu-mediated degradation of CD4 [24]. At least under our experimental conditions, β-TrCP2 was necessary and sufficient to link Vpu to the SCF-E3 ligase complex and to target CD4 for degradation.

### β-TrCP is dispensable required for the HIV-1 release promoting activity of Vpu

**Figure 5:**
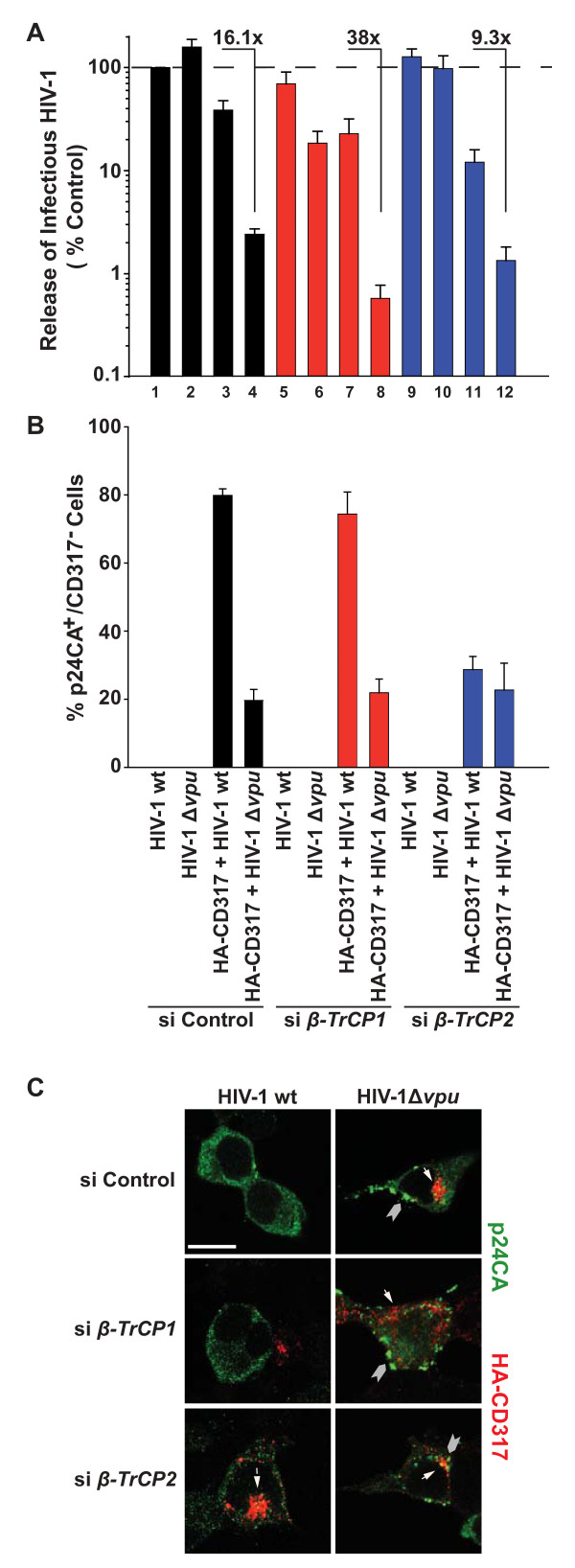
**Vpu cannot degrade CD317 in β-TrCP2-depleted cells, but still enhances HIV-1 release**. 293TCD4 cells were transfected twice with siRNAs targeting either *β-TrCP1 *or *β-TrCP2*, or with a control siRNA, and during the second transfection, plasmids encoding HIV-1wt or HIV-1Δ*vpu*, and HA-CD317 were added. Two days post-transfection, (A) the yield of infectious HIV-1 in culture supernatants was quantified. The arithmetic means ± SEM of six independent experiments are shown. (B, C) Transfected cells from (A) were processed for microscopic analysis to (C) visualize and (B) quantify the percentage of p24CA-positive (green) cells that no longer express HA-CD317 (red). In cells transfected with the specific siRNAs, remaining mRNA levels for *β-TrCP1 *and *β-TrCP2 *were 19 ± 4% and 13 ± 2%, respectively, relative to the control siRNA-treated cells. (B) The arithmetic means ± SEM of three independent experiments are shown. (C) Arrow heads indicate plasma membrane or intracellular p24CA^+ ^aggregates. White arrows indicate HA-CD317 expression in p24CA^+ ^cells. Scale bar: 10 μm.

Next, we investigated the role of both β-TrCP isoforms for Vpu's abilities to promote HIV-1 release and to deplete CD317 (see summary in Table 1). 293TCD4 cells were transfected with isoform-specific *β*-*TrCP-*siRNAs, followed by HIV-1 wt or HIV-1Δ*vpu *proviral DNA in the presence or absence of pHA-CD317. As expected, control siRNA-treated, CD317-expressing cells displayed a strong release restriction for Vpu-defective HIV-1, which was largely overcome by the expression of Vpu in the context of HIV-1 wt (16.1-fold release enhancement, Figure 5A, compare histogram bars 3 and 4). Importantly, this Vpu-dependent release rescue also occurred efficiently in cells that had been depleted for either β-TrCP1 (Figure 5A, histogram bars 7 and 8) or β-TrCP2 (Figure 5A, histogram bars 11 and 12) with a 38- to 9.3-fold virion release enhancement, respectively. Microscopic analysis of these 293TCD4 cells revealed that, in contrast to the scenario in the presence of β-TrCP1ΔF, knockdown of endogenous *β-TrCP2 *abrogated the ability of the provirally encoded Vpu to deplete CD317, resulting in a comparably low fraction of p24CA^+ ^cells that lacked CD317 expression in HIV-1 wt and HIV-1Δ*vpu*-expressing cells (Figure 5B, HIV-1 wt: 29% p24CA^+^CD317^- ^cells. HIV-1Δ*vpu*: 23% p24CA^+^CD317^- ^cells, images in Figure 5C). In contrast, Vpu caused a marked drop in steady-state levels of the restriction factor in both control siRNA- and *β-TrCP1 *siRNA-treated cells (Figure 5B, HIV-1 wt: 74-80% p24CA^+^CD317^- ^cells. HIV-1Δ*vpu*: 20-22% p24CA^+^CD317^- ^cells, images in Figure 5C). These findings suggest that β-TrCP2, but not β-TrCP1, is critical for the Vpu-mediated degradation of CD317. Moreover, neither depletion of CD317 nor the presence of β-TrCP is strictly required for Vpu's ability to promote virus release. Finally, these results establish that the modes of interference with select Vpu functions by β-TrCP1ΔF and by silencing of β-TrCP expression are not equivalent and thus reflect distinct molecular scenarios (see Discussion).

**Figure 6:**
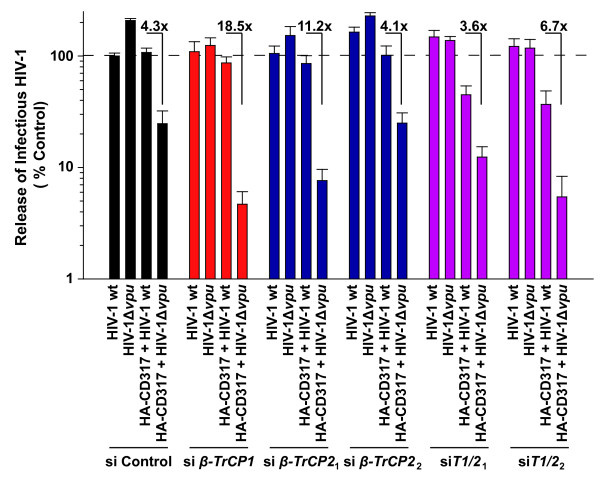
**Co-depletion of β-TrCP1 and β-TrCP2 does not affect Vpu's activity to enhance virus release**. 293T cells were transfected twice with siRNAs targeting either *β-TrCP1 *alone, *β-TrCP2 *alone (with one of two different siRNAs, designated *β-TrCP2*_*1 *_and *β-TrCP2*_*2*_*) *or combinations of siRNAs targeting *β-TrCP1 *and *β-TrCP2 (siT1/2*_*1*_: siRNAs for *β-TrCP1*+ *β-TrCP2*_*1*_, or *siT1/2*_*2*_: siRNAs for *β-TrCP1*+ *β-TrCP2*_*2*_*)*, or with a control siRNA (si Control). During the second transfection plasmids encoding HIV-1wt or HIV-1Δ*vpu*, and HA-CD317 were added. Two days post-transfection, the yield of infectious HIV-1 in culture supernatants was quantified. The factor of difference between the infectious titers for HIV-1 wt and HIV-1Δ*vpu *in cells expressing CD317 is depicted. The arithmetic means ± SEM of three independent experiments are shown.

To exclude the influence of a potential compensatory functional redundancy of the two β-TrCP isoforms in cells in which only one of the isoforms had been depleted, we also performed virion release studies in HA-CD317-expressing 293T cells, in which β-TrCP1 and β-TrCP2 had been depleted simultaneously. The magnitude of Vpu-mediated enhancement in HIV-1 particle release was slightly reduced in these cells when compared to the 293TCD4 cells used before, probably indicating cell type-specific differences. Nevertheless, in all cells expressing HA-CD317, a significant Vpu-dependent rescue of virion HIV-1 release was observed irrespective of their β-TrCP status (Figure 6). The efficiency of *β-TrCP *mRNA depletion ranged between 53-90% (*β-TrCP1 *mRNA) and 81-95% (*β-TrCP2 *mRNA). The increase of infectious virus titers in culture supernatants for the wt compared to the Vpu-defective HIV-1 ranged from 3.6-fold to 18.5-fold and importantly, was seen for two different combinations of siRNAs targeting *β-TrCP1 *and *β-TrCP2 *(Figure 6, si*T1/2*_1 _(3.6-fold enhancement) and si*T1/2*_2 _(6.7-fold enhancement)). These results demonstrate that β-TrCP is dispensable for Vpu's ability to enhance virion release.

### Vpu's ability to rescue HIV-1 release in β-TrCP-depleted cells best correlates with its capacity to reduce surface-exposure, not intracellular levels, of CD317

**Figure 7:**
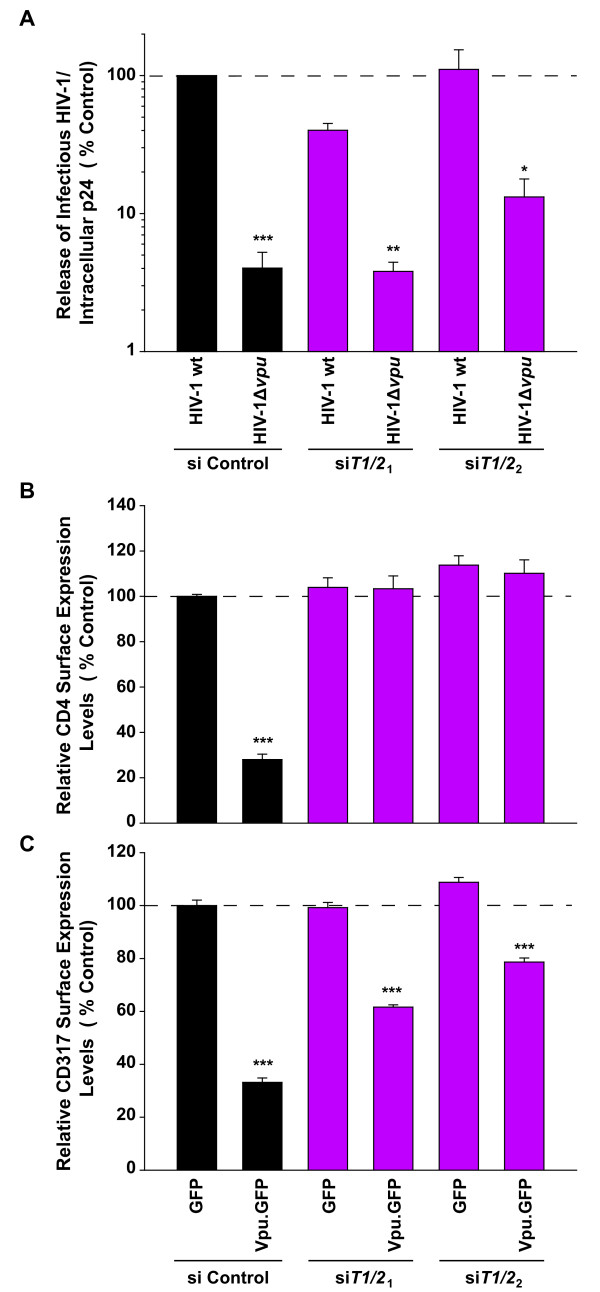
**β-TrCP1/β-TrCP2-depleted TZM-bl cells support Vpu's ability to enhance HIV-1 release and to downregulate endogenous CD317, but not CD4**. TZM-bl cells were transfected twice with combinations of siRNAs targeting *β-TrCP1 *and *β-TrCP2 (siT1/2*_*1*_: siRNAs for *β-TrCP1*+ *β-TrCP2*_*1*_, or *siT1/2*_*2*_: siRNAs for *β-TrCP1*+ *β-TrCP2*_*2*_*)*, or with a control siRNA (si Control). During the second transfection plasmids encoding (A) HIV-1wt or HIV-1Δ*vpu*, or (B, C) GFP or Vpu.GFP were added. Two days post-transfection, (A) the yield of infectious HIV-1 in culture supernatants was quantified and is plotted relative to the cell-associated levels of p24CA with results for HIV-1 wt in control cells given as 100%. The arithmetic means ± SEM of three independent experiments are shown. (B, C) Relative surface levels of CD317 and CD4 on GFP-positive cells were quantified two days post-transfection by flow cytometry. Shown are arithmetic means ± SEM of three independent experiments. Student's-test (comparing results for HIV-1 wt and HIV-1Δ*vpu *for each condition): * p = 0.01, ** p < 0.002, *** p < 10^-4^.

We next assessed the impact of co-depletion of β-TrCP1 and β-TrCP2 in HeLa-derived TZM-bl cells, expressing high levels of CD4 and endogenous CD317. In line with the results observed in 293TCD4 cells with transient HA-CD317 expression (Figure 6), co-depletion of both β-TrCP isoforms did not or only slightly affect the Vpu-dependent rescue of virion release (Figure 7A). In parallel, these siRNA-treated TZM-bl cells were transfected with expression plasmids encoding either Vpu.GFP or GFP alone and 36 hrs later monitored for surface levels of CD4 and CD317. Control siRNA-treated cells showed a marked downregulation of surface-CD4 on cells expressing high levels of Vpu.GFP (28% residual levels, Figure 7B). In contrast, cells treated with both combinations of siRNAs targeting *β-TrCP1 *and *β-TrCP2 *no longer supported Vpu's capacity to downregulate CD4 (Figure 7B). Effects on CD317 surface levels were different: Control siRNA-treated cells supported a strong loss of CD317 from the surface upon Vpu.GFP expression (33% residual levels, Figure 7C), yet cells, in which both β-TrCP isoforms had been co-depleted, still facilitated Vpu's capacity to downregulate surface-exposed CD317. While being highly significant, the degree of Vpu-mediated downregulation in these β-TrCP-depleted cells was reduced compared to control cells (62 to 79% residual levels, Figure 7C).

**Figure 8:**
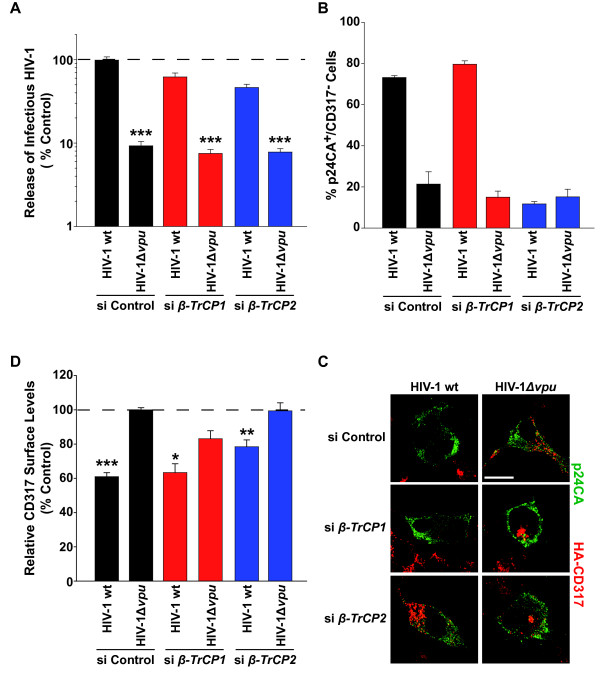
**Vpu antagonism coincides with a reduction of CD317 surface levels, not depletion of intracellular pools of the restriction factor in HIV-infected cells**. 293HA-CD317 cells were transfected twice with siRNAs targeting either *β-TrCP1 *or *β-TrCP2 *or with a control siRNA. Following the second transfection, cells were infected with VSV-G pseudotyped HIV-1 wt or HIV-1Δ*vpu *overnight and washed the following day. Two days post-infection, (A) the yield of infectious HIV-1 in culture supernatants was quantified. The arithmetic means ± SEM of four experiments are shown. (B, C) Half of the infected cultures were processed for microscopic analysis to (C) visualize and (B) quantify the percentage of p24CA-positive (green) cells that no longer express HA-CD317 (red). Scale bar: 10 μm. (B) Shown are arithmetic means of results from two independent experiments. (C) White arrows indicate HA-CD317 expression (red staining) in infected, p24CA-positive (green staining) cells. (D) The other half of the cells was analyzed for surface levels of CD317 on productively infected, p24CA-positive cells by flow cytometry. MFI values for control siRNA-treated cells, infected with Vpu-defective HIV-1, were set to 100%. Remaining cellular mRNA levels for *β-TrCP1 *and *β-TrCP2 *were 23 ± 9% and 3 ± 1%, respectively, relative to the control siRNA-treated cells. The arithmetic means ± SEM (n = 9) from three independent experiments are shown. Student's *t*-test (comparing results for HIV-1 wt and HIV-1Δ*vpu *for each knockdown condition): * p < 0.02, ** p < 0.004, *** p < 10^-5^. In addition, a statistical significance test was performed for results for HIV-1 wt in si Control-treated versus si *β-TrCP2*-treated cells in panel A (p < 0.0001) and panel D (p < 0.0017).

Finally, we sought to study the effects of manipulating individual β-TrCP isoforms on the capacity of Vpu to overcome the virion release restriction and to modulate surface and intracellular levels of CD317 in cells infected with HIV-1. To this end, 293 cells stably expressing HA-CD317 underwent siRNA-mediated depletion of either endogenous *β-TrCP1 *or *β-TrCP2 *and were subsequently infected with vesicular stomatitis virus G protein (VSV-G) pseudotyped HIV-1 wt or HIV-1Δ*vpu*. Two days post-infection, the percentages of productively infected, p24CA-positive cells were similar for all conditions (data not shown). Importantly, regardless of which β-TrCP isoform was depleted (*β-TrCP *mRNA reduction: *β-TrCP1*: 77%; *β-TrCP2*: 97%), the wt virus efficiently overcame the virion release restriction imposed by CD317 (Figure 8A). Similar to cells treated with control siRNA, cell-associated levels of CD317 were still drastically reduced upon depletion of β-TrCP1 in the majority of HIV-1 wt-infected cells (80 ± 2% p24CA^+^CD317^- ^cells versus 15 ± 3% p24CA^+^CD317^- ^cells for HIV-1Δ*vpu *infection; Figure 8B,C). On the contrary, in β-TrCP2-depleted cells, infection by HIV-1 wt failed to affect CD317 pools, compared to the Vpu-defective virus (12 ± 4% versus 15 ± 4% p24CA^+^CD317^- ^cells, respectively, Figure 8B,C). Of particular note, flow cytometric analysis of CD317 surface levels on p24CA-positive cells from the identical cultures demonstrated that the restriction factor was significantly downregulated in a Vpu-dependent manner (Figure 8D), irrespective of cellular levels of β-TrCP1, β-TrCP2, or CD317. Also, the degree of CD317 downregulation was diminished in β-TrCP-depleted cells compared to control cells. We conclude that Vpu's ability to overcome the CD317-mediated virus release restriction in HIV-1-infected cells correlates best with its capacity to reduce surface-exposure, not intracellular levels, of the restriction factor in a β-TrCP-independent manner.

## Discussion

Degradation of CD4 and antagonism of the CD317-imposed virion release restriction have been identified as two cardinal functions of the HIV-1 accessory protein Vpu. Mechanistically, it is well established that Vpu acts as an adaptor between CD4 and the degradation machinery. While binding to CD4 occurs via a hydrophilic C-terminal domain of Vpu [26,33], Vpu is phosphorylated at serine residues 52/56 by casein kinase II [33,34], allowing for recruitment of an E3 ubiquitin ligase multi-protein complex via the substrate recognition factor β-TrCP [21]. Since it is unclear whether Vpu exploits the same general strategy for antagonizing the CD317 restriction to HIV-1 particle release, we used several experimental approaches to investigate the dependence of both major Vpu activities on the di-serine interaction motif of the accessory protein and on expression of cellular β-TrCP (see summary of results in Table 1). We found that β-TrCP2, not the structurally related β-TrCP1, is the critical Vpu adapter for the E3 ubiquitin ligase complex that targets both CD4 and CD317 for accelerated degradation. In contrast, β-TrCP was largely dispensable for Vpu-mediated downregulation of CD317, but not CD4, from the cell surface. Most importantly, β-TrCP was not required for Vpu's ability to counteract the release restriction imposed by CD317.

In agreement with findings in earlier reports [12,14,21,25,26,35], we observed that the integrity of the di-serine motif was strictly required for effects of Vpu on cell-surface exposure and overall expression levels of CD317 and CD4, as well as its antagonism of the virion release block. These results are also in line with a recent report [25] which demonstrated, through the use of a casein kinase II inhibitor, that Vpu phosphorylation is critical for its antagonistic activity. A central role of Vpu's di-serine motif was further supported by findings with β-TrCP1ΔF. This mutant protein binds to this motif [14,27] without coupling the Vpu-substrate complex to the E3 ubiquitin ligase complex. In the context of HIV studies, β-TrCP1ΔF should be regarded as a Vpu inhibitor, since its expression competitively blocks the functionality of the di-serine motif to which β-TrCP and other currently unknown cellular proteins may bind. In agreement with its role as a motif-specific Vpu inhibitor, co-expression of Vpu with β-TrCP1ΔF resulted in a loss of Vpu activities, the pattern of which was similar to that observed with the Vpu_S52/56A _mutant (Table 1). Notably, overexpression of β-TrCP wt, which like β-TrCP1ΔF associates with Vpu [21], did not exert similar inhibitory effects. This discrepancy between overexpression of the F-box-deleted fragment, compared to the full-length protein, possibly reflects different on/off-rates in the interaction of these two molecules with Vpu, governed by the inability of the β-TrCP1ΔF to couple substrate complexes to the E3 ubiquitin ligase complex, possibly resulting in a prolonged occupation of the di-serine motif. Vpu-mediated CD317 depletion in the presence of β-TrCP1ΔF posed a noteworthy exception under our experimental conditions. We speculate that binding of β-TrCP1ΔF specifically impairs the Vpu-mediated proteasomal degradation of CD317 and CD4. While this appears to be the only degradative pathway for CD4, inhibiting this pathway with β-TrCP1ΔF might result in a preferential mistrafficking of CD317 to the *trans*-Golgi network (TGN) or early endosomes, and subsequent delivery to the lysosome [15,16,18].

siRNA-mediated depletion of endogenous β-TrCP provides a direct means for probing its role for Vpu activities. Consistent with two recent reports [14,27], we find that β-TrCP2, but not β-TrCP1, is critical for the depletion of cellular pools of CD317. These reports went on to suggest an inverse correlation of cell-associated CD317 levels and HIV-1 release enhancement by Vpu. To the contrary, we found that depletion of β-TrCP2 or simultaneous depletion of both β-TrCP isoforms did not result in a marked impairment of Vpu's ability to promote HIV-1 release in cells with exogenous and endogenous CD317 expression. Of note, under these conditions, downregulation of CD317 surface levels by Vpu, although statistically highly significant, was less pronounced. The discrepancies between these and our studies may reflect differences in cell lines and experimental conditions. Moreover, some of these previous reports simplified, at times, the interpretation of apparent residual effects of Vpu on virion release rescue in β-TrCP-depleted cells as a lack of functionality of the viral protein [14,16,27]. We included the concomitant validation of *β-TrCP *mRNA levels or side-by-side assessment of Vpu-mediated modulation of CD4 levels following RNA interference in all our β-TrCP depletion studies. This validation together with the robustness of the observed consequences for Vpu activities allowed us to conclude that both β-TrCP isoforms are not strictly required for Vpu counteraction of CD317 in our experimental system and thus are not general Vpu co-factors for this activity.

Based on these findings, we propose an integrative model for Vpu antagonism of CD317-mediated virion release restriction: Vpu binds directly to CD317 [25], involving the transmembrane domains of both proteins [13,26,36]. While the cellular membranes of their primary contact are still undefined, this interaction markedly affects the subcellular distribution of CD317, resulting in a reduction of CD317 surface levels and concomitant accumulation in the TGN (at least in HeLa-derived cells, in which Vpu-mediated degradation is not very prominent). As a downstream consequence of reduced cell-surface exposure of CD317 in the presence of Vpu, CD317 can be subject to accelerated degradation. Two different degradative pathways can apparently be targeted, possibly depending on the specific cellular environment and/or targeted subpopulations of CD317. Proteasomal degradation of CD317 occurs via β-TrCP2 and the E3 ubiquitin ligase complex. Lysosomal degradation may be a consequence of pronounced mistrafficking of CD317 via early endosomes. Importantly, changes in the major intracellular pools of CD317 do not necessarily feed back into the surface population of the restriction factor and are dispensable for Vpu's antagonistic activity. In line with this scenario, Vpu efficiently antagonizes degradation-insensitive CD317 variants, and this correlates with a preserved ability to downregulate the restriction factor from the cell surface [17]. Thus, Vpu-mediated degradation of CD317 can be regarded as a secondary effect of the viral protein on the restriction factor that is not strictly required for its capacity to promote HIV-1 release.

Reduction of cell-surface pools of CD317, therefore, emerges as key activity of Vpu for its role as an antagonist of the virion release restriction. Experimental evidence indicates that Vpu does not enhance the endocytotic rate of surface-exposed CD317 [15], implying that interceptions at the level of anterograde transport of newly synthesized CD317 or of recycling populations of CD317 might underlie the Vpu-mediated downregulation of CD317 from the surface. This activity depends on the phosphorylated di-serine motif in Vpu, but not its *bona-fide *cellular interaction partner β-TrCP2, and thus most likely reflects the interaction of Vpu with another host factor. Reported Vpu-interacting factors include Vpu-binding protein UBP, also referred to as small glutamine-rich tetratricopeptide repeat protein (SGT) [37,38], the MHC II invariant chain CD74 [39] and the TASK-1 ion channel [40]. Since UBP/SGT was dispensable for CD317 antagonism by Vpu (data not shown), most cell types used for studies of Vpu counteraction lack expression of CD74, and TASK-1 interferes with, rather than facilitates Vpu activity, this putative factor still needs to be identified. This factor should bind to the phosphorylated di-serine motif of Vpu and is predicted to mediate the trapping of CD317 at the TGN by direct or indirect mechanisms.

Despite increasing insight into the molecular mechanisms underlying Vpu's ability to overcome the CD317-mediated virion release restriction, key aspects remain unresolved and warrant future investigation. These include the identification of critical cellular Vpu co-factors as well as the definition of relevant intracellular transport steps or subpopulations of CD317 that are affected by Vpu. The emergence of distinct strategies employed by viral factors other than Vpu for counteraction of CD317 [4,5,41-43] emphasizes the potency of this restriction to limit the spread and pathogenesis of viruses.

## Methods

### Cells and plasmids

293T, 293TCD4, 293HA-CD317, TZM-bl, and HeLaP4 cells were maintained in DMEM supplemented with 10% fetal calf serum, 1% penicillin-streptomycin and 1% L-glutamine (all from Invitrogen). 293TCD4 cells were generated by lentiviral vector transduction in principle as reported [44]. 293HA-CD317 cells were generated by transfection of pcDNA3.1-HA-CD317*neo *[12] and neomycin selection. The HA-tag is fused to the N-terminus of CD317. Proviral plasmids pHIV-1NL4-3 wt (BH10 Env) and pHIV-1NL4-3Δvpu (BH10 Env) were from Valerie Bosch [45] and pBR-NL4-3.IRES.eGFPΔ*nef *and pBR-NL4.3-IRES.eGFPΔ*nef*Δ*vpu *[46] from Frank Kirchhoff. The proviral plasmid pHIV-1NL4-3 VpuS52NS56N was generated by site-directed mutagenesis. pcDNA-Vphu, expressing a codon-optimized, Rev-independent HIV-1NL4-3 Vpu protein [47] and pcDNA-VphuS52NS56N were from Klaus Strebel. Analogous expression constructs for Vpu mutants S52A/56A [12], S52A, and S56A were generated by site-directed mutagenesis. pcDNA-Vphu.GFP, encoding a Vpu.GFP fusion protein, was generated by PCR of pSynVphu with the forward primer (5'-CGAATTCTGATGGTGCCCATTATTG-3') and reverse primer (5'-TGGATCCCGCAGGTGGTCAATGTCCCA-3') to generate *EcoRI *and *BamHI *restriction sites and subcloning into pEGFP-N1. HA-tagged and Myc-tagged expression plasmids for human β-TrCP wt and β-TrCP wt ΔF were from Florence Margottin-Goguet [21] and a Flag-tagged expression plasmid for mouse β-TrCP2 wt [48] was from Keiichi Nakayama.

### Transfections

293T cells, 293TCD4 cells (each 1.2 × 10^5^/well) or HeLaP4 cells (1 × 10^5^/well) were seeded in 12-well plates 1 day before calcium-phosphate transfection of proviral DNA (1.2 μg) or empty vector together with pcDNA3.1-HA-CD317*neo *(0.1 μg) and the indicated Vpu expression constructs (0.12 μg). For co-expression of epitope-tagged β-TrCP wt or F-box-deleted fragments, 1 μg of the respective expression plasmids was added. TZM-bl cells (1 × 10^5^/well) were transfected using jetPRIME^® ^(PEQLAB Biotechnologie) according to the manufacturer's protocol.

### HIV-1 infection

293HA-CD317 cells were infected with VSV-G HIV-1_NL4-3 _wt (BH10 Env) or isogenic Vpu-defective virus at a multiplicity of infection of 0.2.

### Virus release quantification

The virion- and cell-associated amount of HIV-1 p24CA antigen was determined by an antigen enzyme-linked immunosorbent assay (p24CA ELISA) [49]. The infectivity of HIV-1 in culture supernatants was determined 2 days after transfection on TZM-bl reporter cells as reported [50].

### Surface-exposed receptor levels

To quantify CD4 expression at the cell surface, washed cells were stained in PBS with allophycocyanin (APC)-conjugated mouse-anti human CD4 antibodies (clone RPA-T4; BD Bioscience). To quantify CD317 surface-exposure, cells were stained with mouse anti-HM1.24/CD317 [51] (5 μg/ml; Chugai Pharmaceuticals) followed by staining with goat anti-mouse (Alexa Fluor 660) (Invitrogen). A parallel assessment of the productive infection was performed after fixing cells with 4% paraformaldehyde (PFA) for 90 min by staining for intracellular p24CA antigen with Fluoresceinisothiocyanat (FITC)-conjugated KC57 monoclonal antibodies (Beckman Coulter) in 0.1% Triton X-100/PBS for 30 minutes on ice. A FACS Calibur with BD CellQuest Pro 4.0.2 Software (BD Pharmingen) was used for analysis. The MFI for surface-exposed receptors was quantified in principle as reported [52].

### Western blotting

Washed cell pellets were lysed in SDS-lysis buffer. Proteins were separated on 12.5% SDS-PAGE and blotted to nitrocellulose membranes. Blocked membranes were probed with the following primary antibodies: mouse monoclonal anti-HIV-1 p24CA antibody 183, rabbit polyclonal anti-Vpu antisera (from Ulrich Schubert or Klaus Strebel), anti-MAPK antiserum (Santa Cruz Biotechnology), mouse anti-HA mAb HA.11 (Covance), mouse anti-c-Myc antibody (Santa Cruz Biotechnology) and mouse-anti Flag M2 antibody (Sigma-Aldrich). Secondary antibodies were conjugated either to horseradish peroxidase for ECL-based detection or to Alexa Fluor 700/800 fluorescent dyes for detection by Odyssey Infrared Imaging System (LI-COR Biosciences) and quantification by Odyssey software (version 2.1).

### Immunofluorescence microscopy

Transfected or infected cells growing on coverslips were fixed with 4% PFA and permeabilized for 2 min with 0.1% Triton X-100 in PBS. Cells were blocked for 1 hour with 1% bovine serum albumin in PBS and stained with appropriate primary and secondary antibodies for detection of p24CA, HA, CD4, or CD317, respectively. Coverslips were mounted in mounting medium (Dianova) and analyzed with a Zeiss LSM510 confocal microscope with a 100x PLAN-APO objective lens. Images were recorded with the Zeiss proprietary software LSM5 and processed with Adobe Photoshop 6.0. Gag localization was grouped for individual cells into marked accumulation (at the plasma membrane as well as intracellularly (cells with at least one p24CA^+ ^aggregates) or diffusely cytoplasmic), in principle as reported [2,12,17,43]. Effects of Vpu on cellular expression of CD4 or CD317, respectively, was judged by comparing to untransfected/uninfected neighbouring cells, scoring cells as displaying reduced expression levels when the fluorescent signal was unambiguously lower.

### siRNA-mediated β-TrCP depletion

Seeded cells were transfected twice on consecutive days by calcium-phosphate precipitation or jetPRIME with siRNAs specific for *β-TrCP1 *mRNA (5'-GAAUUCACUUAGAC AGACA-3'), *β-TrCP2 *mRNA (5'-AGAUUAUCCAGGAUAUAGA-3' (*β-TrCP2*_*1*_), and 5'-GAAGUAAAUCGACCG UCAA-3' [24] (*β-TrCP2*_*2*_)), combinations of siRNAs specific for *β-TrCP1 *and *β-TrCP2 *mRNA, or nonspecific control siRNA (5'-AGGUAGUGUAA UCGCCUUG-3') (each 50 pmol) and harvested 48 hours after the second transfection. Where indicated, proviral DNAs or expression plasmids for CD317, Vpu.GFP or GFP were co-transfected, or cells were infected with VSV-G HIV-1 at the second time-point.

### β-TrCP knockdown quantification

Total RNA was extracted by a standard Trizol-chloroform protocol and precipitated with isopropyl alcohol. RNA pellets were washed with 75% ethanol, dissolved in water, and stored at -80°C until use. After treatment with *DNase *DNA-free (Ambion) and cDNA synthesis (NEB), relative quantitative PCR analyses were performed on the ABI Prism 7500 sequence detection system (Applied Biosystems). *β-TrCP *mRNA levels were quantified with primers specific for *β-TrCP1 *and *β-TrCP2*, reported [24], and TaqMan-specific probes for *β-TrCP1 *(5'-FAM-GCAAGCACTGCTATGAAGAC-TAMRA-3') and *β-TrCP2 *(5'-FAM-CAGTCTGCACTT TCACCCGT-TAMRA-3'), respectively. *β-TrCP *mRNA levels were quantified by using the 2^-ΔΔCt ^method with human RNaseP gene as endogenous reference control. Pooled triplicates were analyzed for each condition. Data analysis was conducted using the 7500 System Software (Applied Biosystems).

## Competing interests

The authors declare that they have no competing interests.

## Authors' contributions

HMT, SH, OTF and OTK designed the study. HMT, SH, JVF and IA conducted the experiments. OTF and OTK wrote the paper. All authors commented on and approved the final manuscript.

## Supplementary Material

Additional file 1 **HIV-1-encoded VpuS52NS56N cannot counteract endogenous CD317**. The provirally expressed di-serine mutant of Vpu cannot overcome the CD317-imposed virion release restriction in TZM-bl cells. Click here for file

Additional file 2 **Expression of HA-CD317, Vpu wt, or its serine mutants, does not affect expression or maturation of HIV-1 Gag**. Expression neither of CD317/Tetherin nor Vpu considerably alters the expression levels or processing of the Gag polyprotein of HIV-1 in 293T cells. Click here for file
