# Supplementary material for: β-TrCP is dispensable for Vpu's ability to overcome the CD317/Tetherin-imposed restriction to HIV-1 release
Source: Retrovirology. 2011 Feb 10;8:9. doi: 10.1186/1742-4690-8-9 (PMC3049139; doi:10.1186/1742-4690-8-9)
Supplement: Additional file 1 — HIV-1-encoded VpuS52NS56N cannot counteract endogenous CD317. The provirally expressed di-serine mutant of Vpu cannot overcome the CD317-imposed virion release restriction in TZM-bl cells. [file 1742-4690-8-9-S1.PDF]

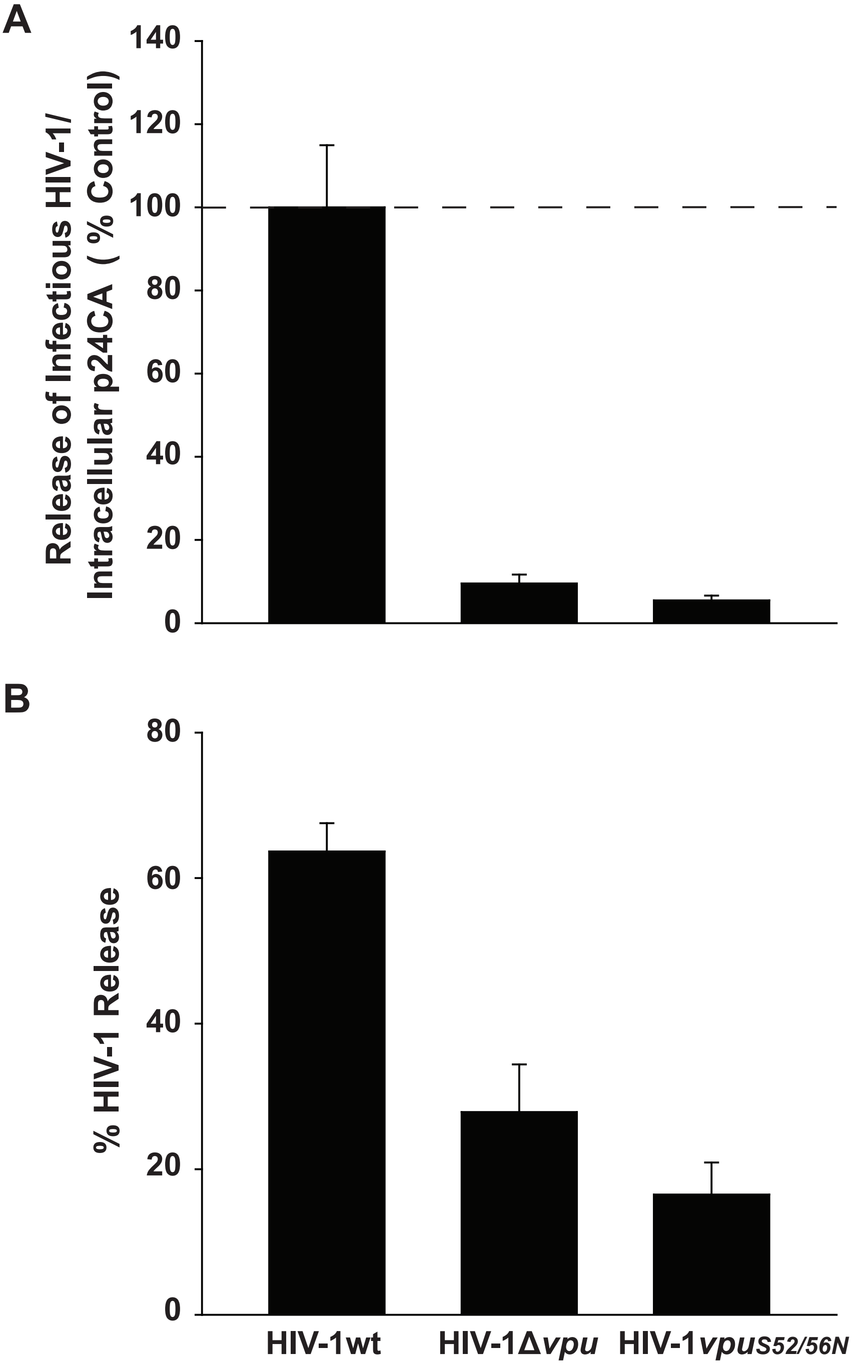

Figure S1

HIV-1-encoded VpuS52NS56N cannot counteract endogenous CD317. TZM-bl cells were transfected with plasmids encoding HIV-1wt, HIV-1Δ*vpu*, or HIV-1*vpu*$_{S52NS56N}$. Two days post-transfection, (A) the yield of infectious HIV-1 in culture supernatants was quantified in a standard infectivity assays and the values were normalized to cell-associated levels of p24CA, determined by p24CA ELISA.The arithmetic means ± SD (n = 12) are shown. (B) Data from the same experiments are presented as HIV-1 release expressed as the percentage of total p24CA (in cells and supernatant) that was secreted as virion-associated p24CA, in principle as reported [12]. The arithmetic means ± SD (n = 12) are shown.
