# Supplementary material for: β-TrCP is dispensable for Vpu's ability to overcome the CD317/Tetherin-imposed restriction to HIV-1 release
Source: Retrovirology. 2011 Feb 10;8:9. doi: 10.1186/1742-4690-8-9 (PMC3049139; doi:10.1186/1742-4690-8-9)
Supplement: Additional file 2 — Expression of HA-CD317, Vpu wt, or its serine mutants, does not affect expression or maturation of HIV-1 Gag. Expression neither of CD317/Tetherin nor Vpu considerably alters the expression levels or processing of the Gag polyprotein of HIV-1 in 293T cells. [file 1742-4690-8-9-S2.PDF]

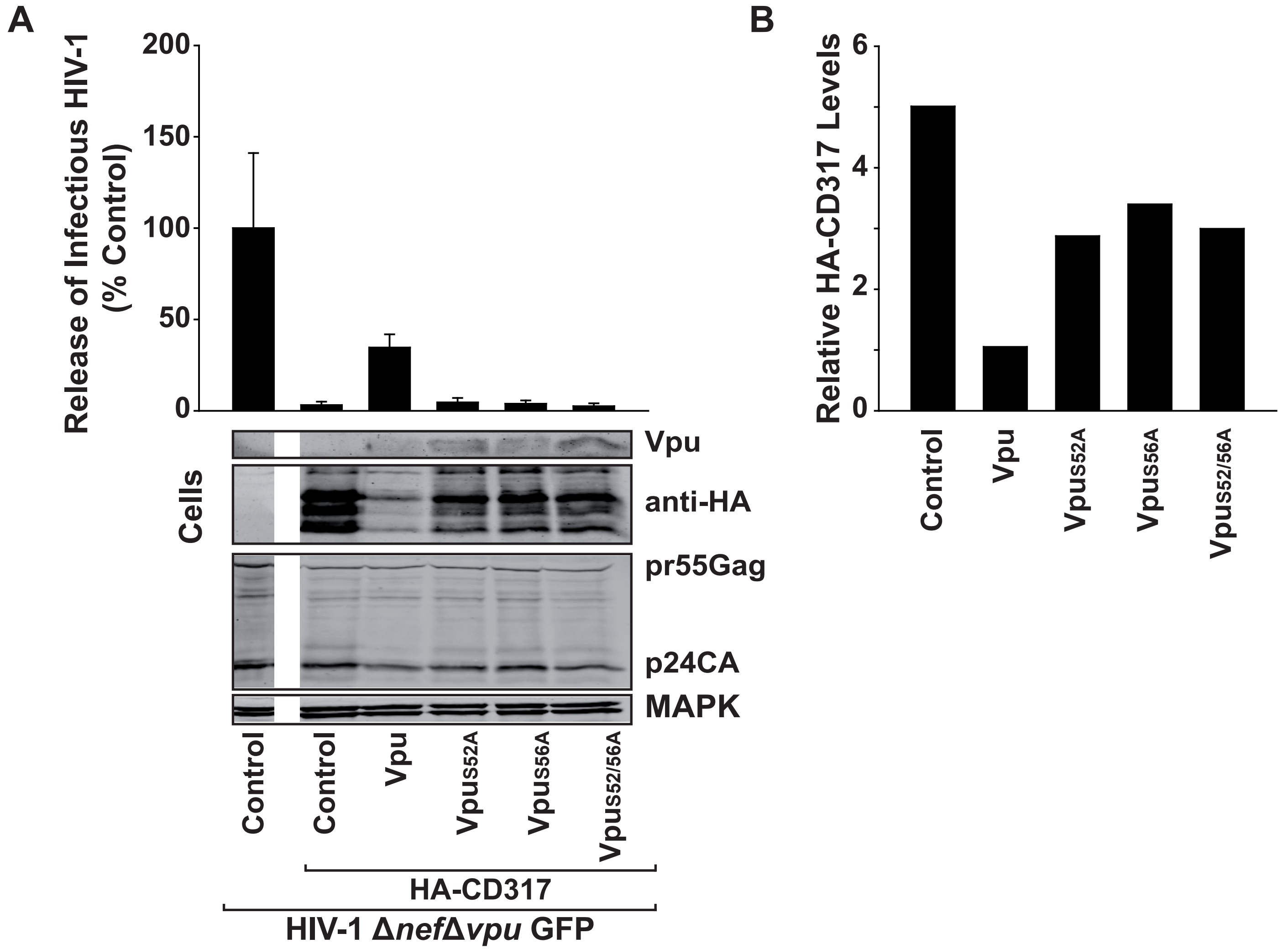

Figure S2

Expression of HA-CD317, Vpu wt, or its serine mutants, does not affect expression or maturation of HIV-1 Gag. 293T cells were transfected essentially as described in the legend to Fig. 1. (A) Two days post-transfection, the yield of infectious HIV-1 in the supernatant and cell-associated levels of Vpu, HA-CD317, p24CA, and MAPK were analyzed by titration on TZM-bl indicator cells and by western blotting, respectively. The HIV-1 yields are plotted relative to the condition in the absence of HA-CD317 (Control), which was set to 100%. Shown are arithmetic means + SD (n=6) from one of two independent experiments. Western blots shown represent samples run on the identical gel with the gap indicating an area where non-informative lanes were omitted. (B) Cell-associated CD317 levels relative to MAPK are given in arbitrary units after quantification of the western blots shown in A.
